# Enhancement effect of AgO nanoparticles on fermentative cellulase activity from thermophilic *Bacillus subtilis* Ag-PQ

**DOI:** 10.1186/s43141-023-00619-1

**Published:** 2023-11-29

**Authors:** Saddam Hussain, Muhammad Talha Yasin, Khurshid Ahmad, Suleman Khan, Rasheed Ahmad, Jallat Khan, Abdul Ghani, Muhammad Musaddiq Shah, Muzzamil Ahmed, Hasnat Tariq, Hamid Rehman, Adil Hussain, Muhammad Faheem, Syed Ali Imran Bokhari

**Affiliations:** 1 Department of Biological Sciences, International Islamic University, Islamabad, 44000 Pakistan; 2 Xinjiang Key Laboratory of Biological Resources and Genetic Engineering, College of Life Science and Technology, Xinjiang University, Urumqi, Xinjiang 830046 China; 3 Institute of Biological Sciences, Khwaja Fareed University of Engineering and Information Technology, Rahim Yar Khan, 64200, Pakistan; 4 State Key Laboratory of Marine Food Processing & Safety Control, College of Food Sciences and Engineering, Ocean University of China, No. 1299, Sansha Road, Qingdao, Shandong Province 266404 P.R. China; 5 Department of Physics, NFC Institute of Engineering and Technology, Multan, 60000 Pakistan; 6 Department of Chemical Engineering, University of Engineering & Technology (UET), Peshawar, Khyber Pakhtunkhwa 25120 Pakistan; 7 Department of Biological Sciences, Faculty of Sciences, University of Sialkot, Sialkot, Punjab 51040 Pakistan; 8 Department of Environmental Sciences, Quaid-i-Azam University, Islamabad, 45320 Pakistan; 9 Food and Biotechnology Research Centre (FBRC), Pakistan Council of Scientific and Industrial Research (PCSIR), Laboratories Complex, Ferozepur Road, Lahore, 56400 Pakistan

**Keywords:** Cellulase, Nanocatalyst, *Bacillus subtilis*, AgO FeO NPs, Industrial applications

## Abstract

**Background:**

Cellulase is an important bioprocessing enzyme used in various industries. This study was conducted with the aim of improving the biodegradation activity of cellulase obtained from the *Bacillus subtilis* AG-PQ strain. For this purpose, AgO and FeO NPs were fabricated using AgNO_3_ and FeSO_4_·7H_2_O salt respectively through a hydro-thermal method based on five major steps; selection of research-grade materials, optimization of temperature, pH, centrifuge, sample washed with distilled water, dry completely in the oven at the optimized temperature and finally ground for characterization. The synthesized NPs were characterized by scanning electron microscope (SEM), energy dispersive X-ray (EDX), and X-ray diffraction (XRD) to confirm the morphology, elemental composition, and structure of the sample respectively. The diameter of the NPs was recorded through SEM which lay in the range of 70–95 nm.

**Results:**

Cultural parameters were optimized to achieve better cellulase production, where incubation time of 56 h, inoculum size of 5%, 1% coconut cake, 0.43% ammonium nitrate, pH 8, and 37 °C temperature were found optimal. The enhancing effect of AgO NPs was observed on cellulase activity (57.804 U/ml/min) at 50 ppm concentration while FeO NPs exhibited an inhibitory effect on cellulase activity at all concentrations. Molecular docking analysis was also performed to understand the underlying mechanism of improved enzymatic activity by nanocatalysts.

**Conclusion:**

This study authenticates AgO NPs as better nanocatalysts for improved thermostable cellulase biodegradation activity with the extraordinary capability to be potentially utilized in bioethanol production.

**Graphical Abstract:**

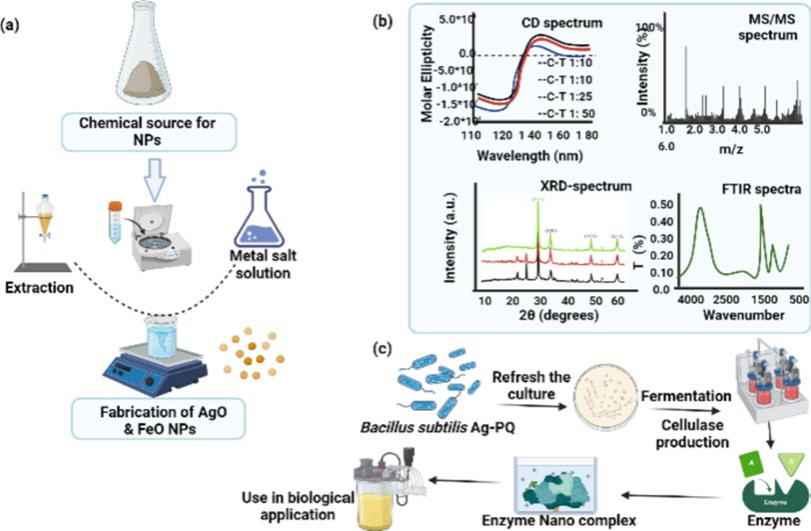

## Background

Carboxymethyl cellulase (CMCase) belongs to a class of cellulase enzymes known as endo -1, 4- β-glucanase, which can convert cellulase into soluble glucose [1]. Microbial cellulase has a number of applications in various industries, including the pulp and paper industry, textile industry, bioethanol industry, wine and brewery industry, food processing industry, animal feed industry, detergent industry, waste management, and agriculture industry [2]. Cellulase is an inducible enzyme that is synthesized by a wide variety of microorganisms including fungi and bacteria, and they can be anaerobic, aerobic, thermophilic, or mesophilic [3]. The costs of these enzymes produced by fungi are very high and therefore bacteria are preferred because they have a short generation time and high growth rate as compared to fungi [4, 5]. Bacteria are successful candidates for the industrial production of cellulase enzymes because they have the capability to produce cellulases that can tolerate extreme conditions such as acid, alkali, and thermostability [6]. The majority of these bacteria are found and isolated from animal waste, compost, soil, and sugar cane [7]. Various bacteria are reported for the production of cellulase such as *Bacillus* sp. including *B. brevis*,* B. cereus*,* B. amyoliquefaciens* DL-3*, B. subtilis* YJ1,* B. vallismortis* RG-07, *B. megaterium*, *B. pumilus*, and *B. circulans* [1, 8] Gram positive *B. subtilitis* has been extensively studied in past years [9]. They are well characterized and have remarkable fermentation properties and a high yield of production. They also produce non-toxic by-products and have high adaptability to environmental changes [10, 11]. Previous studies have reported the production of CMCase from *Bacillus subtilitis* [12–14].

Cellulosic biomass is composed of lignin, cellulose, and hemicellulose, which is one of the most abundant renewable resources [15]. Cellulosic biomass can be transformed into a variety of products, including paper and pulp, animal feed and textiles, using various technologies, and in particular Biotechnology. The abundant and renewable cellulosic biomass also represents a potential alternative resource to fossil fuels, with rising global demands for energy [16]. Cellulase (EC 3.2.1.4) first hydrolyzed cellulosic biomass to glucose for conversion into other products, and then different biological or chemical processes could be performed. The degradation of cellulosic material has therefore received considerable attention for improved cleaning process [17]. Different methods for hydrolyzing cellulase have been proposed, including steam explosion, acid-activated montmorillonite catalysts, alkaline, acid, enzymatic hydrolysis, and microbiological methods [18, 19]. In nature, the enzyme-mediated cellulase breakdown requires various types of cellulolytic enzymes, the major ones being β-1,4-exoglucanase (EC 3.2.1.91), β-1,4-endoglucanase (EC 3.2.1.4), and β-glucosidase (EC 3.2.1.21) [20]. The challenges to industrial applications of these enzymes are high cost and low production yield that need to be overcome with improved cellulase production [5, 21]. One of the limiting factors for biofuel production from cellulose feedstocks is the inefficient transformation of cellulase into fermented sugars. New ways of enhancing the kinetics and stability of cellulases are crucial to the economic feasibility for production of biofuel [22].

Metal ions have the ability to interact with a carboxylic acid or amine group of amino acids and in this way, they activate or inhibit the activity of the enzyme [1, 5]. Several studies have shown the role of metal ions in the activation or inhibition of microbial cellulases [23–25]. Nanoparticles can play a significant role in improving the pH and thermal stability of the cellulase enzymes due to various unique chemical and physical characteristics [26] such as high surface reaction activity, large surface-to-volume ratio, strong adsorption ability, and high catalytic efficiency [18, 19]. In industry, nanoparticles have proven to be beneficial catalysts [26]. Several studies have reported the use of nanoparticles to enhance the production, activity, pH, and thermal stability of cellulose [18, 19]. Biosynthesized silver nanoparticles were used as nanocatalysts and showed a two-fold increase in the cellulose degradation activity of cellulase [26]. Carboxymethyl cellulose (CMC) assay revealed an increase in the activity of carboxymethyl cellulase (CMCase) in the presence of CaCl2 nanoparticles [27].

By considering the industrial importance of cellulase, the presented study was conducted for the production and characterization of cellulase *Bacillus subtilis* AG-PQ. Cultural parameters were optimized to achieve maximum cellulase yield by *Bacillus subtilis* AG-PQ strain using solid-state fermentation and submerged fermentation. Further, the study explored the silver and iron NPs effect on the biodegradation activity of cellulase. This study will help understand NPs’ interaction with cellulase for its beneficial and effective role in various industries such as paper and pulp, food, and textile industries.

## Methods

This study was conducted in the Applied Microbiology and Biotechnology Lab (AMBL) at the International Islamic University, Islamabad, Pakistan. All the chemicals used in this study were of analytical grade.

### Synthesis of silver oxide and iron oxide nanoparticles by hydrothermal method

A hydrothermal technique was adopted to synthesize silver oxide and iron oxide nanoparticles (NPs) for our desired morphology at the nanoscale. The advantage of such a technique over others was its ability to make the desired NPs in a pure crystalline phase in large quantities. Such NPs in pure and controlled size were obtained by adjusting the factors that affect the morphology of NPs during the experimental process like temperature, pressure, and reactants, also research-grade equipment played a vital role that was used during the hydrothermal method; Furnace, Oven, Teflon, Autoclave, Centrifuge Machine, digital balance, magnetic stirrer and mortar, and pestle [28, 29]. To synthesize pure crystalline of required NPs, the hydrothermal technique is based upon six major steps; (i) research-based materials selection; (ii) optimization of molarity and pH of the solution; (iii) precursors were mixed uniformly through magnetic stirrer; (iv) adjust temperature for the reaction; (v) reaction proceed and pressure sustained in the autoclave; and (vi) centrifuge the sample and completely dried in an oven [30, 31]. To study and improve the biodegradation activity of cellulose we synthesized silver oxide and iron oxide NPs using a hydrothermal technique. For such a project, research-grade salt 1 M of AgNO_3_ was selected and dissolved in 50 ml distilled water. The precursor AgO was prepared by the addition of 2 M aqueous solution of NaOH dropwise with vigorous constant stirring (1300 rpm) on a hot plate at 60 °C for 1 h. Then continuous precipitate of AgO was synthesized till the reaction stopped at pH = 11.4. The resulting colloidal mixture was put into Teflon. The Teflon was shifted and placed into the stainless-steel autoclave. Then the autoclave was kept in a conventional oven for 24 h at 180 °C. Subsequently, the colloidal were shifted to the test tube to centrifuge for 2 h. After centrifuge, the sample (supernatant) was washed with distilled water a couple of times and also washed a minimum of two times with ethanol to remove the impurities from the NPs. The resultant product was dried in an oven for 5 h at 100 °C. The product was then ground for 30 min. At the end of grinding the sample was ready for characterization [32]. The iron oxide NPs were synthesized in a similar manner as explained above for silver oxide NPs. For such NPs, the research-grade salt 1 M FeSO_4_·7H_2_O was selected and dissolved into 50 ml distilled water. The AgO and FeO NPs were characterized by scanning electron microscope (SEM), Energy Dispersive X-ray (EDX), and X-ray diffraction (XRD). The morphology and elemental compositional were determined by using *Hitachi SU6600* scanning electron microscope (SEM), and energy dispersive X-ray spectroscopy (EDX). The structural composition was confirmed from *Panalytical X-pert pro MPD* X-ray diffraction (XRD).

### Sampling and isolation of microorganisms

Healthy soil samples were collected from the Agriculture region (Latitude 33^o^ 64′ 98′, Longitude 73° 03′ 02′) of Islamabad, Pakistan in sterilized glass bottles and transported aseptically to a laboratory for further processing. The samples were diluted up to 10^−10^ and suspended on an agar plate supplemented with 0.5% peptone, 0.5% yeast extract, Tris–HCl buffer of pH 9, and NaCl with 2% agar [30]. The inoculated plates were incubated at 37 °C. Each distinct colony was picked and sub-cultured again on above mentioned supplemented agar plates to obtain a pure culture. Morphological and biochemical characterizations were performed to identify the isolated bacterium and further screened for cellulase activity by using submerged fermentation [15].

### Morphological, biochemical, and molecular identification

The isolated strain was observed under the light microscope (LM) with 50–100 × resolution. Morphological and physiological characteristics of bacterial strain were examined based on shape, color, respiration, pH, and optimum temperature for proper identification [31]. Different biochemical tests including oxidase test, catalase test, Simmon citrate test, urease test, Voges Proskauer test, methyl red test, indole test, hydrogen sulfide, acid formation from sugars, and OF (oxidation/fermentation) test were performed to identify bacterial strain [31–33]. For molecular identification, the genomic DNA was isolated by phenol–chloroform method and the 16S rRNA region was amplified by PCR using universal 27f and 1492r primer pair [34]. The amplified region was sequenced commercially (Macrogen Korea). Subsequently, the raw sequenced data were assembled and analyzed with BioEdit version 7.1.9 [35]. The sequence was submitted to the NCBI database under the accession number MG662180. Phylogenetic analysis was carried out by using the obtained sequence with previously published 16S rRNA sequences of *Bacillus* sp. retrieved from GenBank (*n* = 31). The multiple sequence alignment was analyzed to construct a maximum likelihood tree with 1000 bootstrap replicates using the software MEGA-7 [36].

### Enzyme and protein assay

The spectrophotometric assay performed to quantify the cellulase activity with a cellulose substrate using the method given by Miller [37]. The reaction mixture consisted of 0.5 ml of a crude enzyme, 0.5 ml of 0.5% cellulose, and 1 ml of 50 mM sodium phosphate (pH) 8.0 and was subjected to incubation at 50 °C for 20 min. After the incubation period was over, 1 ml of Dinitrosalicylic acid (DNS) was added to terminate the reaction. The mixture was boiled for 5 min and the optical density (OD) was measured at 540 nm with a spectrophotometer (UV-1700 APC). Bradford method [38] was used to assess the protein content and the obtained results were accessed by measuring absorbance in a spectrophotometer (UV-1700 APC) at 590 nm.

### Optimization of the time course, medium, and inoculum size for cellulase production

One factor at a time approach was used for the optimization of cellulase production from *Bacillus subtilis* AG-PQ. Four different inoculum concentrations (2.5%, 5%, 7.5%, and 9%) were prepared and added into a 250-ml Erlenmeyer flask containing 100 ml production media. Five different types of media were tested to optimize the best fermentation medium for the maximum yield of cellulase. All these media were inoculated with optimized culture conditions. All experiments were performed in triplicate and bars displayed mean and S.D.

### Effects of culture conditions on cellulase production

Economic carbon sources such as agricultural waste were utilized to make the cellulose production process cost-effective. The effect of economic carbon sources such as rice husk, corn cob, cellulose, wheat bran, and coconut cake at 2% concentration was examined for improved cellulase production [39]. Different nitrogen sources such as urea, ammonium sulfate, diammonium phosphate, ammonium chloride, and ammonium nitrate were added to the fermentation medium to observe their effect on cellulase production [40]. The pH of the production medium was adjusted to 3–8 by using 0.1 N sodium phosphate buffer and 0.1 N citric acid Vyas et al. [41] to investigate the influence of pH on cellulose yield. In order to examine the effect of temperature, fermentation was carried out at temperatures ranging from 25 to 40 °C to optimize the best-suited temperature for cellulase production.

To evaluate the effect of silver (Ag) and iron (Fe) nanoparticles on CMCase production from isolated strains, various concentrations of these nanoparticles (10 to 100 ppm) were added to the fermentation media. The enzyme assay and protein estimation were performed after every 8 h interval for quantification of cellulase activity and protein content.

### Partial purification and characterization of cellulase enzyme

The ammonium sulfate precipitation (APS) method was used for the partial purification of the enzyme according to the method described by Gomori, [42]. The crude extract was treated with different concentrations of APS (20%, 30%, 40%, 50%, 60%, 70%, 80%, 90%) to obtain the purified fraction of cellulase [43]. The precipitates were dissolved in 10 mM phosphate buffer (pH 8) and suspended in a dialyzing membrane. This membrane dialyzed against the same and was kept in a refrigerator at 4 ℃ for 24 h. Various concentrations of cellulose (substrate) were added to the reaction mixture to examine the effect of substrate concentration on cellulase activity [43, 44]. The effect of pH and temperature on cellulase activity was determined at various pH values (4–9) and temperatures (30–65 °C). To investigate the effect of silver oxide (AgO) and iron oxide (FeO) nanoparticles on cellulase activity, various parts per million (ppm) concentrations (10–70 ppm) of these nanoparticles were added to the reaction mixture [45]. Lineweaver–Burk plot was plotted to calculate the *K*_m_ and *V*_max_ values.

### Molecular docking sides of Ag and Fe nanoparticles with cellulase activity

To predict the preferred binding sites and binding mode of Ag and Fe with cellulase enzyme Auto Dock Vina tool was used by Trott and Olson [46]. The structures of Ag and Fe were obtained from PubChem (https://pubchem.ncbi.nlm.nih.gov). The 3D structure of CMCase (PDB ID: 3PZU) was obtained from Protein Data Bank https://www.rcsb.org/structure/3PZU. Before docking analysis, water molecules were removed from the protein. To determine the binding sites on protein, Ag, and Fe molecules were allowed to move within the whole protein region. The output from AutoDock Vina was further analyzed with PyMOL (Ahmad et al. [5] used for the structural representation of figures.

## Results

### Synthesis and characterization of silver and iron oxide NPs

**Fig. 1:**
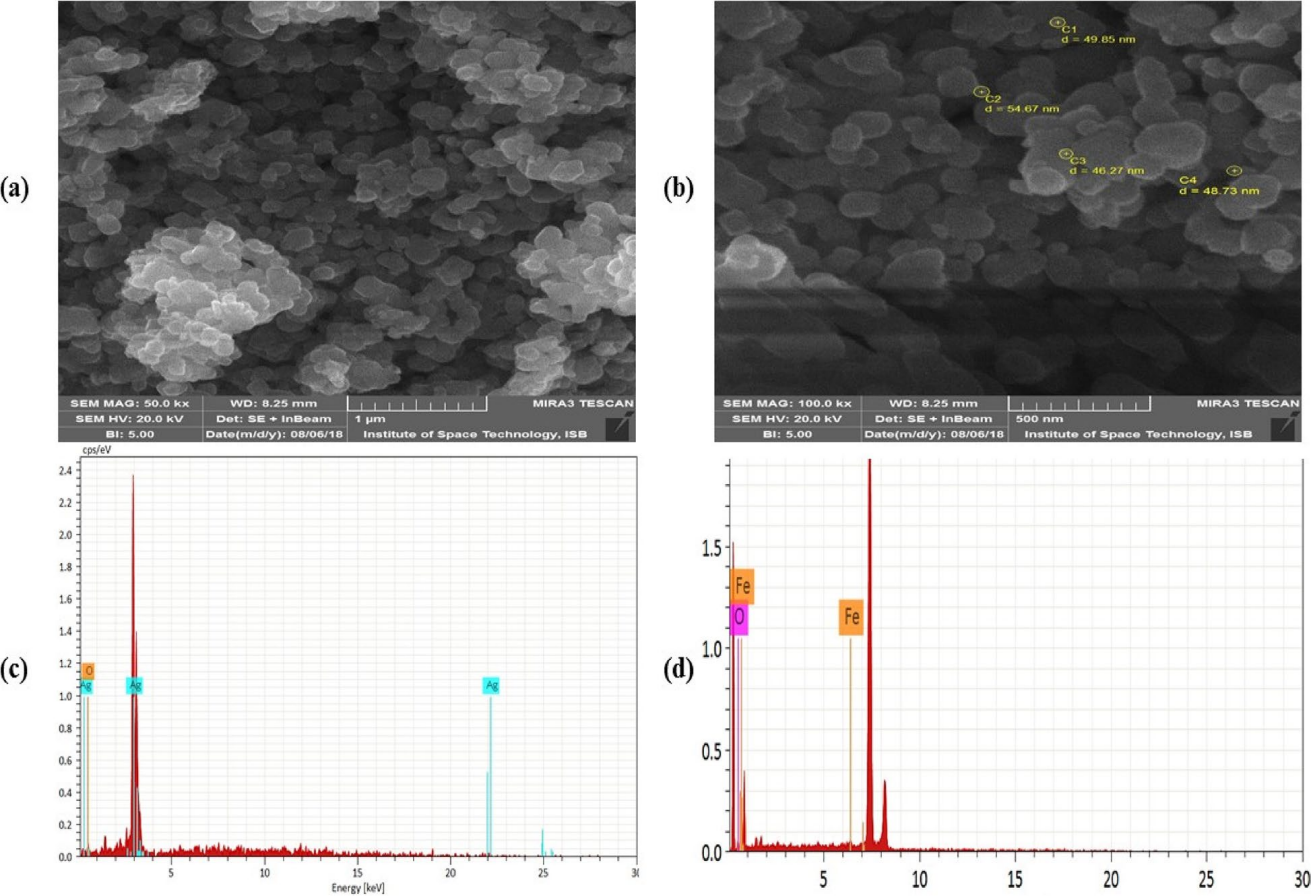
SEM and EDX micrographs of nanoparticles. **a** SEM micrograph of silver oxide (AgO). **b** SEM micrograph of iron oxide (FeO). **c** EDX micrograph of (AgO) nanoparticles. **d** EDX micrograph of (FeO) nanoparticles

The morphology and elemental composition of synthesized silver and iron oxide nanoparticles (NPs) examined through a scanning electron microscope (SEM) and energy dispersive X-ray analysis (EDX) are shown in Fig. 1, where surface morphology confirming the hexagonal and circular shape of silver oxide and iron oxide nanoparticles is evident (Fig. 1a, b). The impurities-free NPs with smooth uniform grain dispersions and surfaces can be seen. The diameter recorded for these NPs was in the range of 70–95 nm. The elemental composition of these nanoparticles was confirmed through EDX as shown in Fig. 1c, d. The different peaks are also shown in Fig. 1c, d because the gold was sputtered on top of NPs as a conducting material before SEM characterization.

### Morphological, biochemical, and molecular identification of bacterial strain

The isolated bacterium was identified as *Bacillus subtilis* based on morphological, biochemical, and molecular characterization, and the strain was designated as AG-PQ. The data of morphological and biochemical tests for the strain *Bacillus subtilis* AG-PQ (MG662180) is presented in Table 1.

**Table 1:** Identification of *Bacillus subtilis* strain on the basis of biochemical tests in comparison with *B. subtilis* BTN7A

S/N	Biochemical test	*Bacillus subtilis* AG-PQ	*Bacillus subtilis* TP4-2	*Bacillus subtilis* BTN7A	*Bacillus* sp.38b
1	Gram nature	Gram positive	Gram-positive rods	Gram-positive rods	Gram-positive rods
2	Form	Irregular	Irregular	Irregular	Irregular
3	Surface	Rough	Rough	Rough	Rough
4	Catalase	** + **	** + **	** + **	** + **
5	Oxidase	** + **	** + **	** + **	** + **
6	Motility	** + **	** + **	** + **	** + **
7	Citrate utilization	** + **	** + **	** − **	** − **
8	Lactose	** − **	** − **	** − **	** − **
9	H_2_S production	** + **	** + **	** − **	** − **
10	Methyl red	** + **	**-**	** + **	** + **
11	Voges proskauer	** + **	** + **	**-**	** + **
12	Indole production	** − **	** − **	** − **	** − **
13	Urease	** − **	** − **	** − **	** − **
14	Mannitol	** + **	** + **	** + **	** + **

**Fig. 2:**
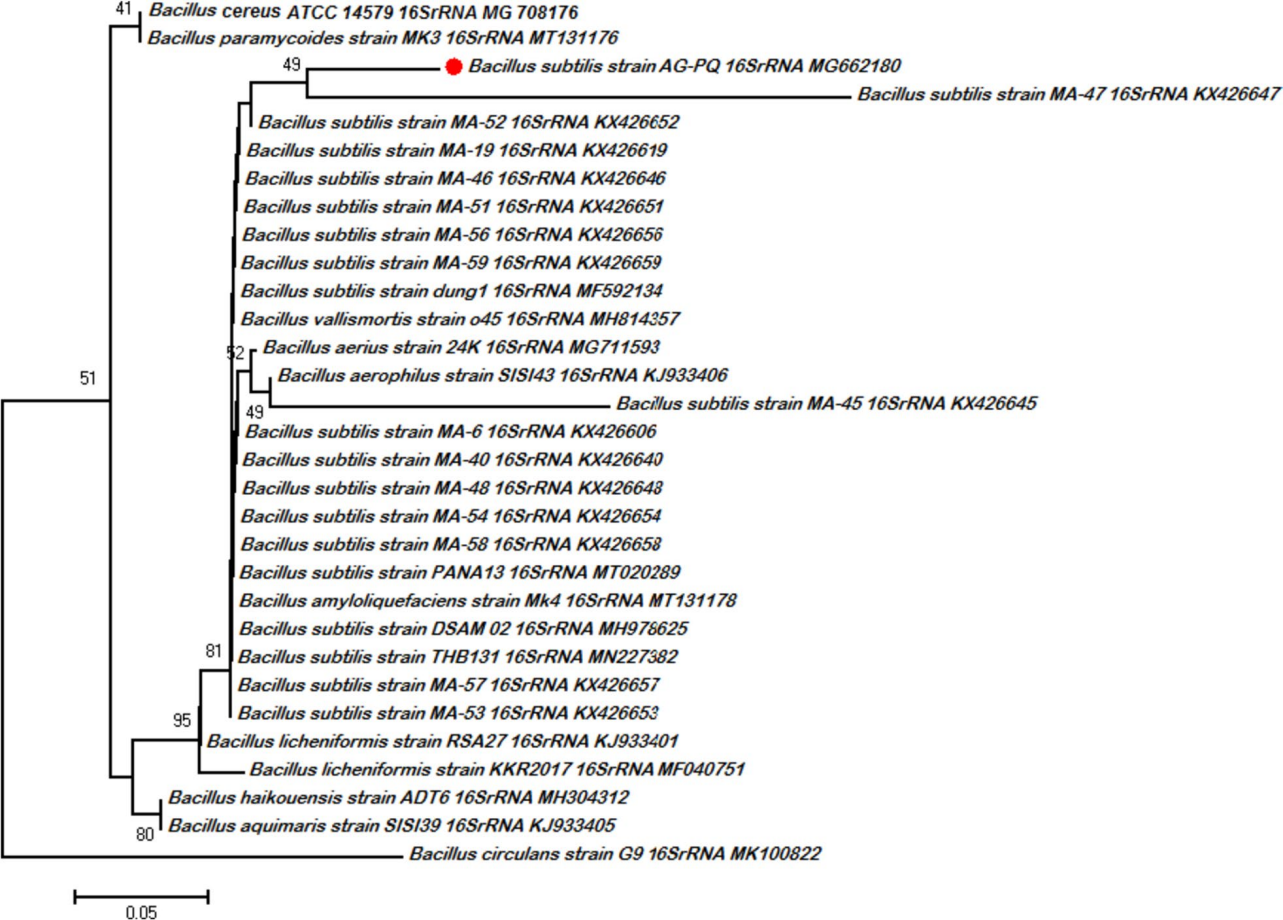
Neighbor-joining consensus tree based on partial 16S rDNA gene sequences showing the evolutionary relationships between *Bacillus subtilis* AG-PQ of the present study (Specified in red) with reference strains from GenBank. The significance of each branch is indicated by the bootstrap value calculated for 1000 replicates (only values higher than 50% are indicated)

The amplified 16S rRNA region of *Bacillus subtilis* genomic DNA, ~ 1000 base pairs. The data presented in the NJ tree (Fig. 2) based on the 16S rRNA gene shows the dispersion of isolated bacterial strains throughout the clades corresponding to other bacterial strains. The tree indicated that all the bacterial strains form a well-supported group.

### Effects of cultural parameters on cellulase production

**Fig. 3:**
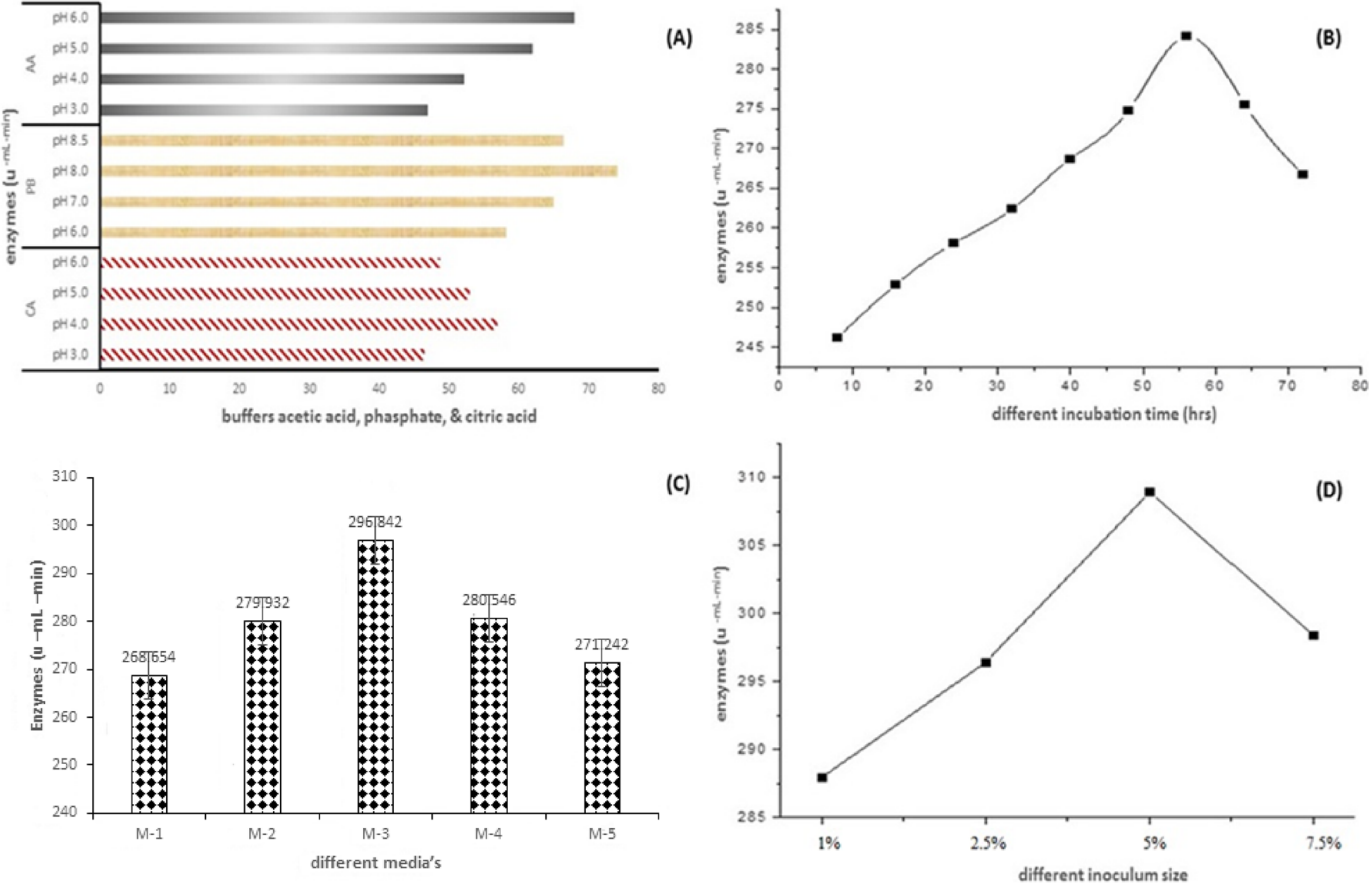
Optimization of cultural parameters on production of cellulase by *Bacillus subtilis* AG-PQ. **a** Buffer optimization. **b** Effect of incubation time on cellulase enzyme production. **c** Production media’s optimization. **d** Effect of inoculum size on cellulase enzyme production

The effect of critical cultural parameters was optimized that can influence the cellulase production by *B. subtilis* AG-PQ using submerged fermentation, the optimization of culture media composition according to the Ahmad et al. [5] design of experimental methodology. The production medium for cellulase allowed incubating for 72 h to optimize incubation time. The maximum cellulase production was achieved after 56 h of incubation time (Fig. 3B). Therefore, 56 h is designated as the optimal time for cellulase production for subsequent experimentation. The results are presented in Fig. 3. It is evident from the results that the optimal inoculum size for cellulase production was 5% at which the maximum 315.0 U/ml/min enzyme activity was observed. The cellulase activity declined to (312.0 U/ml/min) at 7.5% inoculum concentration.

Four different media were used for the optimization of the fermentation process to achieve better enzyme activity. The basal media was found optimal for efficient cellulase activity (302.5 U/ml/min) at 37 °C and 56 h of incubation time (Fig. 3).

**Fig. 4:**
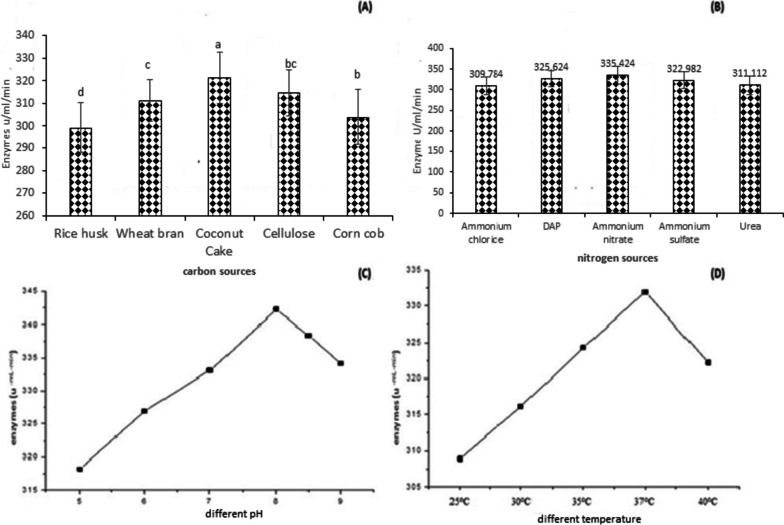
Optimization of different sources for the production of cellulase enzyme. **a** Carbon source. **b** Nitrogen source. **c** pH. **d** Temperature

Five different carbon sources, i.e., rice husk, cellulose, wheat bran, corn cob, and coconut cake, were investigated to opt for the optimal carbon source for cellulase production. The results of this study designated coconut cake as the best carbon source for optimal cellulase production with values of 321.0 U/ml/min in contrast to cellulose (318.0 U/ml/min) and wheat bran (316.0 U/ml/min) (Fig. 4a). Ammonium sulfate, ammonium chloride, urea, ammonium nitrate, and diammonium phosphate were supplemented as nitrogen sources in the production media to achieve the maximum cellulase yield. The results of this study validate ammonium nitrate as the best nitrogen source for cellulase production with the highest values of 329.0 U/ml/min followed by DAP (325.0 U/ml/min) and ammonium sulfate (326.0 U/ml/min) (Fig. 4b).

The pH of the production medium was adjusted to various pH ranges from 5 to 9 to analyze the influence of pH on cellulase production by *Bacillus subtilis* AG-PQ MG662180. Cellulase production was observed maximum (330.0 U/ml/min) at pH 8. The effect of temperature on the production of cellulase was observed by incubating the production media at different temperatures ranging from 20 to 60 °C. The maximum cellulase production (336.0 U/ml/min) was recorded at 37 °C.

The various parts per million (ppm) of silver oxide and iron oxide nanoparticles were supplemented into an optimized fermentation medium to examine the effect on cellulase production. The maximum cellulase production was noticed at a 50-ppm concentration of AgO NPs (336.0 U/ml/min). It has been also observed that Fe NPs particles pose adverse effects in cellulase production with the lowest units recorded at 100 ppm (320.0 U/ml/min).

### Partial purification and characterization of cellulase enzyme

**Fig. 5:**
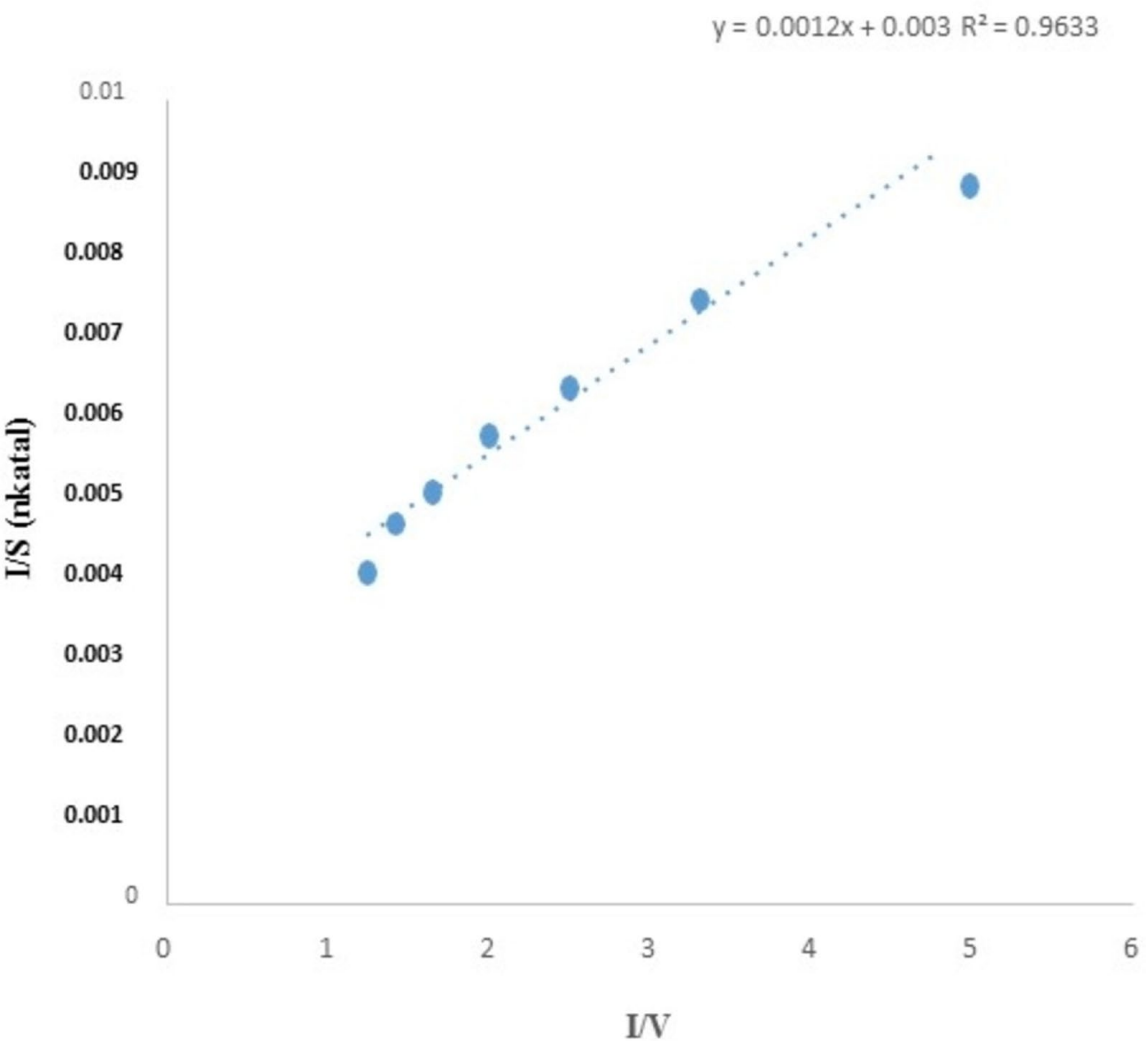
Lineweaver–Burk plot of enzyme activity showing a linear order increase in enzyme activity

Cellulase was partially purified from the crude extract by ammonium sulfate precipitation (ASP) at 50%. The maximum cellulase activity was observed at 50% ammonium sulfate concentration (360 U/ml/min). Kinetic parameters of partially purified cellulase were determined by incubating the enzyme at various cellulose concentrations (0.1–0.9 mM) to analyze the effect of substrate concentration on enzyme activity under standard assay conditions. Cellulase showed maximum enzyme activity at 0.9 mM substrate concentration (286.4 U/ml/min) in the reaction mixture (Fig. 5).

**Fig. 6:**
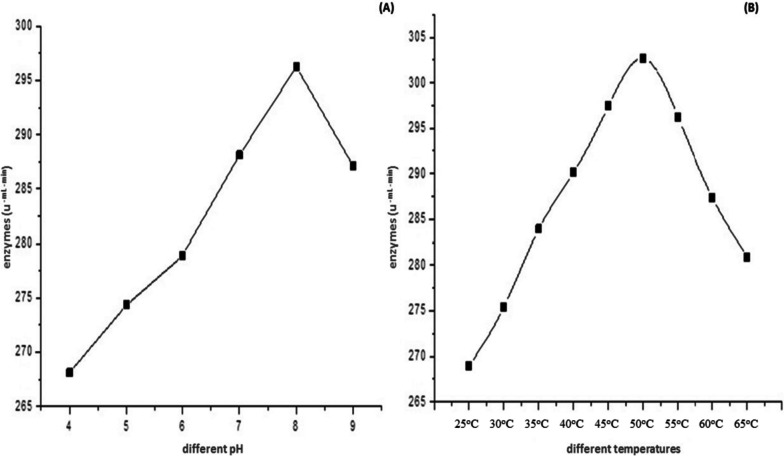
**a** Effect of various pH on cellulase enzyme activity. **b** Effect of various temperatures on cellulase enzyme activity

The dependency on the pH of cellulase activity was investigated by varying pH values ranging from pH 4–9. Cellulase exhibits optimal activity (323.0 U/ml/min) at pH 8. Various temperature ranges from 25 to 65 °C, were evaluated under standard conditions to observe thermal stability and optimal cellulase activity (Fig. 6a, b). The cellulase activity was found maximum at 50 °C.

### Effect of AgO and FeO NPs on purified cellulase

The partially purified cellulase was investigated against several concentrations of AgO and FeO nanoparticles (10–70 ppm) to observe their positive or inhibitory effect. Results showed that AgO nanoparticles have positively influenced cellulase activity and improved thermal stability over a period of time (Table 2). The enzyme activity (57.804 U/ml/min) progressively enhanced up to 25 ppm concentration whereas the enzyme activity started decreasing significantly with an increase in AgO NPs onwards.

**Table 2:** Effect of silver oxide (AgO) and iron oxide (FeO) NPs on purified cellulase enzyme activity

Nanoparticles Parts per million (ppm)	AgO NPs + Enzyme U/ml/min	FeO NPs + Enzyme U/ml/min
0	48.145	48.145
10	49.242	47.469
20	51.06	45.991
30	53.43	43.051
40	55.932	42.425
50	57.804	39.353
60	54.304	35.383
70	50.970	32.736

FeO nanoparticles posed a negative inhibitory effect on cellulase activity. The enzyme activity at 10 ppm concentration was 47.469 U/ml/min and decreased intensely to 32.736 U/ml/min at 70 ppm.

### Docking interaction of Ag and Fe nanoparticlas with cellulase

**Fig. 7:**
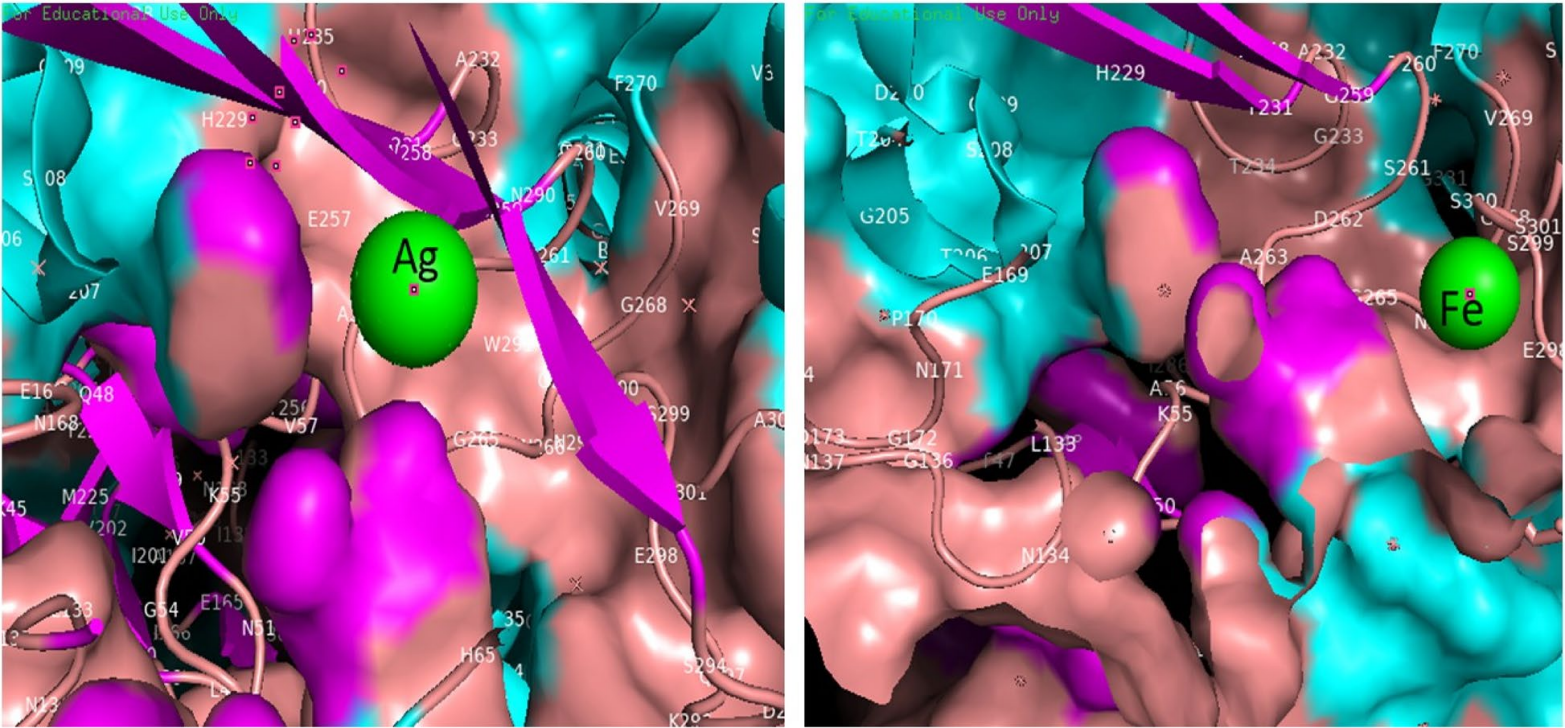
3D structure of *B. subtilis* AG-PQ protein in ribbon presentation. Silver and iron molecules/ligands (shown in green color)

Molecular docking analysis revealed that a list of ligands with their binding energy or binding affinity to understand the possible interaction of different structures with target molecules molecular docking method is very effective. It gives useful information about protein–ligand interaction. In this study, molecular docking was carried out using Auto Dock Vina to find binding sites of AgO and FeO. The results revealed that AgO nanoparticles had − 0.49 kcal/mol binding energy and FeO had − 0.27 kcal/mol binding energy (Fig. 7). Ag nanoparticles had more affinity for surviving compared to Fe nanoparticles because the lower the binding energy more stable the complex.

## Discussion

**Fig. 8:**
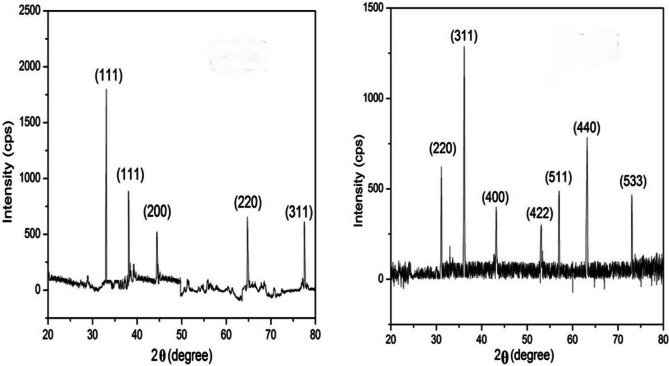
X-ray diffraction pattern of synthesized silver oxide (AgO) NPs and iron oxide (FeO) NPs

The crystal structure of the synthesized nanoparticles (NPs) of silver and iron oxide were analyzed through the XRD method using the Debye Scherrer formula $=\frac{{\varvec{k}}{\varvec{\lambda}}}{{{\varvec{\beta}}}_{{\varvec{h}}{\varvec{k}}{\varvec{l}}}{\varvec{cos}}{\varvec{\theta}}}$ that was exposing Cu-Kα (1.5406$\overset{\circ}{\mathrm{A}}$) in the radiation of incident X-rays wave length $(\lambda )$. The diffracted intensities of both NPs were measured with respect to 2θ, which range was taken from 20° to 80° as shown in Fig. 8, where $\theta$ is the Braggs angle, $k$ indicates the shape factor or correction factor $(k=0.9)$ and ${\beta }_{hkl}$ represent FWHM (full width at half maximum) that was taken in radians [47]. The XRD pattern of Ag NPs was observed at 2θ = 33.07°, 38.07°, 44.44°, 64.75° and 77.47° which correspond to the Miller indices (111), (111), (200), (220), and (311) planes respectively. Such results were confirmed and compared with standard data (JCPDS: 89–3722 and 04–0783) that reflect a face-centered-cubic (FCC) crystal structure corresponding to crystalline in nature [XRD-Ag-1, XRD-Ag-2]. The phase identification of iron oxide (Fe_2_O_3_) NPs displaying their peaks at 2θ = 31.12°, 36.14°, 43.19°, 53.09°, 57.02°, 63.22°, and 73.04° are assigned to the planes (220), (311), (400), (422), (511), (440), and (533). The patterns of these NPs were compared with standard data (JCPDS: 82–1533 and 39–1346) [XRD-FeO-1, XRD-FeO-2]. The EDX clearly shows the presence of oxygen with iron confirming the purity crystal of iron oxide NPs (Fig. 1D). Also in both silver and iron oxide NPs, some brooding diffraction peaks were observed that also confirming their small size and crystalline nature with no other phase of impurity in both patterns of XRD.

*Bacillus subtilis* AG-PQ of this study appeared in a clade comprising other bacterial strains of the *Bacillus* genus and was found in the same lineage with previously reported *Bacillus subtilis* strains as shown in Fig. 2. Morphological and biochemical characterization provides authentic information about the isolated unidentified strain and its extra/intracellular secretions but molecular characterization is a reliable approach to identifying the isolated strain through phylogenetic analysis by using 16S rRNA sequences [48]. The incubation time was observed and cellulase production was decreased by further increasing the incubation period. This might be due to the exhaustion of nutritional components or the production of secondary metabolites in the production medium [39]. Reddy et al. [49] reported that the optimal incubation time for cellulase production by *Bacillus subtilis* was 60 h. Whereas, Kiran et al. [39] showed that the optimal incubation time for the production of cellulase by *Bacillus subtilis* was 48 h. The results of Yan et al. [9] revealed that the cellulase production by the *Bacillus subtilis* Q-3 strain increased with an increase in incubation time and reached its maximum production at 60 h.

Inoculum size plays a crucial role in the optimization of the fermentation process [50]. To optimize the inoculum size concentration, production media was inoculated with different inoculum concentrations (2.5%, 5%, 7.5%, and 9.0%) to observe their effect on cellulase production. Similar results were reported by Shajahan et al. [51] who optimized the 5% inoculum concentration for the highest cellulase production by *Bacillus subtilis*. Whereas, Singh and Kaur [52] reported that 5% inoculum size was best for maximum cellulase production by *Bacillus subtilis* Q-3 strain. Similar observations were made by Hussain et al. [32] for media optimization who reported the maximum production of cellulase using basal media production medium by *B. subtilis*.

Enzyme regulation is majorly influenced by the culture media composition during the fermentation process [39]. Previous literature reported that carbon and nitrogen sources affect cellulase production from microbial sources [6]. Also, Sethi et al. [40] found coconut cake to be the best inducer for the production of cellulase enzymes by *B. subtilis*. Soeka [53] reported the maximum production of cellulase from *B. subtilis* A8 by utilizing rice bran and corncob as substrate. Vyas et al. [41] also reported ammonium nitrate as the best source of nitrogen for cellulase production by *B. subtilis*. Sethi et al. [40] tested various nitrogen sources, among which ammonium sulfate was found to be the best source of nitrogen for cellulase production by *B. subtilis*. Zhou et al. [54] reported optimized cellulase production from *Bacillus subtilis* MU S1 by keeping the pH of the medium constant at pH 8. Similarly, Zamani et al. [55] observed maximum cellulase activity by *B. subtilis* at pH 7. Hussain et al. [32] showed maximum cellulase activity by *Bacillus sp.* 313SI under stationary conditions at 37 °C. In another study, 37 °C temperature was found to be optimal for cellulase production by *Bacillus subtilis* Q-3 Yan et al. [9]. Otajevwo and Aluyi [56] demonstrated a significant ability of *B. subtilis* SBMP4 strain to produce cellulase at 37 °C.

Lineweaver–Burk plot was plotted against the obtained data in order to retrieve *K*m and *V*max values [57]. Anu et al. [54] optimized pH 8 for the maximum activity of partially purified cellulase. Similar observations were made by Kiran et al. [39] who reported the maximum cellulase activity at 50 °C from *Bacillus subtilis*. Cellulase exhibits stability over a range of temperatures with moderately stable at alkaline pH ranges.

Kumar et al. [36] investigated the effect of FeO NPs on the soil microbial community and reported iron oxide nanoparticles as enzyme inhibitors. The results are in concurrence with the observations of Salunke et al. [26] who investigated the effect of biosynthesized Ag nanoparticles on fungal cellulase activity to enhance cellulase degradation. Here, a two-fold increase was observed in enzymatic activity with Ag nanoparticles in cellulose degradation. The results of this study confirm that the combination of free thermostable cellulose and AgO NPs is effectively used for significant cellulose degradation.

Another study by Shah et al. [58] and Eisazadeh et al. [59] examined the effect of Ag NPs on hydrolytic enzymes such as lipase and cellulase to be potentially used in the detergent industry. They revealed that Ag NPs affect the secondary structure of lipase and cause the reduction in its catalytic activity whereas loss of anti-microbial activity and stability of Ag NPs was reported upon interaction with the lipase. Cellulase has been influenced by Ag-NPs in comparison to lipase and its catalytic activity was significantly changed suggesting the positive correlation of Ag-NPs with cellulase. Recently, Gupta et al. [60] reported that biosynthesized Ag NPs have a positive influence on fungal cellulase catalytic activity, stability, and biocompatibility. In our study, cellulase activity was significantly enhanced in the presence of Ag NPs (10–50 ppm), and the cellulase activity was boosted two-fold at 0.012 µg/ml/min of Ag NPs concentration. Srivastava et al. [19] evaluated the pH and thermal stability of cellulase in the presence of chemically synthesized Zinc oxide (ZnO) nanoparticles. Cellulase showed alkaline stability at pH 10.5 and retained 53% activity whereas the enzyme remained thermally stable at 65 °C for 10 h. The results of this study signify the importance of nanocatalysts for enhanced enzymatic activity to be potentially used in cellulase degradation industries.

## Conclusions

Agricultural soil is an abundant source of useful biocatalysts and other valuable substances. *Bacillus subtilis* AG-PQ can efficiently produce cellulase of sufficient quantity by consuming coconut cake as a sole carbon source and ammonium nitrate as a nitrogen source with solid-state fermentation (SSF). Other cultural parameters such as pH and temperature were also optimized to obtain the maximum cellulolytic activity. This study has successfully utilized silver oxide nanoparticles for enhancing the production and activity of cellulase from *Bacillus subtilis* AG-PQ. Improved cellulase production and biodegradation activity were demonstrated by cellulase in the presence of silver oxide nanoparticles. The study indicates that *Bacillus subtilis* AG-PQ could be a better candidate for industrial processes owing to its thermophilic nature and thermostable cellulase production.

## Data Availability

Not applicable.

## Abbreviations

µl: Microliter
NPs: Nanoparticles
AgO: Silver oxide
FeO: Iron oxide
µ: Micro
SEM: Scanning electron microscopy
EDS: Energy-dispersive X-ray spectroscopy
XRD: X-ray Diffraction Analyzer
ml: Milliliter
min: Minutes
AMBL: Applied Microbiology and Biotechnology Lab
M: Molar
NaOH: Sodium hydroxide
h: Hours
SSF: Solid state fermentation
OD: Optimum density
